# Activity of Arylsulphatase in Soil Contaminated with Polycyclic Aromatic Hydrocarbons

**DOI:** 10.1007/s11270-014-2097-4

**Published:** 2014-08-06

**Authors:** Aneta Lipińska, Jan Kucharski, Jadwiga Wyszkowska

**Affiliations:** Department of Microbiology, University of Warmia and Mazury in Olsztyn, Plac Lodzki 3, 10-727 Olsztyn, Poland

**Keywords:** PAHs, Organic substances, Arylsulphatase, Resistance, Resilience

## Abstract

An experiment has been performed to determine the activity of arylsulphatase in soil submitted to pressure of four polycyclic aromatic hydrocarbons: naphthalene, phenanthrene, anthracene, and pyrene, in the amount of: 0, 1,000, 2,000, and 4,000 mg kg^−1^ dm of soil. Soil samples were also applied some organic substances, such as: cellulose, sucrose, and compost, in the amount of 0 and 9 g kg^−1^ dm of soil. The experiment was run under laboratory conditions. It was established on soil which belonged to loamy sand. The soil resistance (RS) and resilience (RL) indices were computed. It has been discovered that the PAHs stimulated arylsulphatase activity, with anthracene raising the activity of the enzyme to the highest degree. The activity of arysulphatase depended significantly on the dose of a PAH, duration of pressure, and type of organic substances added to soil. The highest resistance (RS) was determined in soil exposed to phenanthrene, and the lowest one—in soil polluted with pyrene. Low values of the RL index prove that polycyclic aromatic hydrocarbons cause lasting disorders in the activity of arylsulphatase.

## Introduction

Polycyclic aromatic hydrocarbons belong to organic compounds with strong toxic, mutagenic, and carcinogenic properties. Their distinguishing feature is a variety of structural forms composed of two or more benzene rings, depending on a hydrocarbon. PAHs can accumulate in the environment, and their persistence in particular compartments of the environment is governed by their concentration, dispersion of these compounds, pH, oxygen content, and availability of nutrients (Bamforth and Singleton 2005; Li et al. 2010; Wyszkowski and Ziółkowska 2013). Most PAHs (about 90 %) are accumulated in soil, which receives PAHs from combustion of wood and fossil fuels, emission of fumes from motor vehicles, or soil application of sewage sludge (Kwach and Lalah 2009; Maliszewska-Kordybach et al. 2009). Despite such high accumulation of PAHs in soil, it is possible to remove these contaminants with increasingly advanced methods, including physicochemical ones, e.g., extraction with solvents (Khodadoust et al. 2000), chemical, e.g., photocatalytic degradation (Zhang et al. 2008), electrokinetic remediation (Reddy et al. 2006), or oxidation on a chemical (Gan et al. 2009), thermal (Falciglia et al. 2011), or biological pathway (Lu et al. 2011). Among the latter methods, next to phytoremediation, most attention is paid to bioremediation, as it is perceived to be a safe and economically viable method. The aim of bioremediation is to catalyze and transform organic pollutants, including the ones less harmful to the environment. This is a natural way to break down polycyclic hydrocarbon with microorganisms such as bacteria, fungi, and algae (Bamforth and Singleton 2005).

An important role in bioremediation technologies is performed by biostimulation, i.e., enhancement of the development of autochthonous microorganisms by introduction of specific nutrients or growth stimulators (e.g., sucrose, compost, or cellulose) into soil. Apart from biostimulation, there is also bioaugmentation, where selected strains of microorganisms are introduced to the environment in order to accelerate the rate of decomposition of PAHs. Bioremediation processes can be used worldwide to such aims as improved soil fertility by introduction of multi-culture vaccines in areas polluted by PAHs. The rate of PAH biodegradation depends on physicochemical and biological parameters, including counts and diversity of soil microorganisms, and on conditions in which organic compounds are decomposed, i.e., availability of nutrients, presence of oxygen, pH, and temperature. The changeability of temperature could be one of the deciding factors in attaining improved efficiency of biodegradation. Antizar-Ladaslao et al. (2007) demonstrated experimentally that the highest percentage of removed PAHs during a composting process was achieved at the constant temperature of 38 °C, but it was 70 °C that tuned out to be the best bioremediation option. In turn, Delille et al. (2007) conducted an experiment on Antarctic, in which they demonstrated that even a small rise in temperature (by 2.2 °C) may improve the capability of microorganisms to decompose PAHs.

Due to frequent and uncontrolled leakage of petroleum substances on the premises of pumping stations, petrol stations, and petroleum processing plants, the soil environment is penetrated by large quantities of polycyclic aromatic hydrocarbons (Shrestha et al. 2010). These considerations have given rise to the experiment presented herein, in which doses of naphthalene, phenanthrene, anthracene, and pyrene several-fold higher than naturally occurring ones have been tested.

The tendency of PAHs to bind to soil organic matter and on the surface of particles is directly correlated with the number of benzene rings in a hydrocarbon molecule (Maliszewska-Kordybach 2005; Anderson 2013); hence, the longer the hydrocarbon chain, the lower the bioavailability of PAHs as substrates for microorganisms. This and some other factors are responsible for disturbing the biological parameters of soil, including the most important ones such as counts of microorganisms and enzymatic activity, which enable analysts to define the direction and intensity of organic and mineral transformations in the soil environment. Arylsulphatase, assayed in the present experiment, is a sulfate enzyme with broad distribution in nature (Elsgaard et al. 2002), which catalyzes the release of SO_4_^2−^ from sulfate esters. Arylsulphatase plays a role of an indicator of sulfur mineralization in soil and also has an important part in the cycling of this element. Arylsulphatase is found intra and extracellularly; thus, its activity needs to be assessed in both of these forms (Vong et al. 2008). In a study by Klose and Tabatabai (1999), 45 % of the determined pool of arylsulphatase was extracellular and 55 % occurred in live cells of microorganisms.

The objective of this study was to determine the effect of soil contamination with naphthalene, phenanthrene, anthracene, and pyrene on the activity of arylsulphatase. Another objective was to make an attempt to alleviate the influence of the PAHs on arylsulphatase by amending soil with organic substances, such as cellulose, sucrose, and compost. Finally, based on the activity of arylsulphatase, the indices of soil resistance and resilience were calculated.

## Materials and Methods

### Soil

The soil material used in the discussed experiment had the texture of loamy sand, in which the sand (0.05–2 mm), silt (0.002–0.05), and loam fractions (<0.002 mm) made up 72.42, 25.31, and 2.27 % of soil bulk, respectively. The soil was sampled from an arable field located at the experimental station in Tomaszkowo (Poland). Samples were collected from the topsoil at a depth of 0–20 cm. Air-dry soil was passed through a 2-mm-mesh sieve. The soil material had the following characteristics: pH_KCl_, 7.1; hydrolytic acidity, 4.8 mmol(+) kg^−1^; total exchangeable bases, 279 mmol(+) kg^−1^; and organic carbon content, 13.1 g kg^−1^; total nitrogen content, 1.1 g kg^−1^; content of available phosphorus, 200 mg kg^−1^; potassium, 147 mg kg^−1^; and magnesium, 27 mg kg^−1^.

### Polycyclic Aromatic Hydrocarbons

The following PAHs were investigated: naphthalene, phenanthrene, anthracene, and pyrene. They were added to soil samples in the form of loose-weighed amounts: 0, 1,000, 2,000, and 4,000 mg kg^−1^ dm of soil. The reason why naphthalene, phenanthrene, anthracene, and pyrene were chosen for the study as representatives of PAHs is their importance in soil remediation, recognized, for example, by the US Environmental Protection Agency (US EPA). All the four PAHs are lipophilic and very difficult to dissolve in water. The *n*-octane/water partition coefficient for naphthalene originating from the B&K Multi-branch Company, and for phenanthrene, anthracene, and pyrene supplied by Acros Organics was 3.6, 4.4.6, 4.5, and 4.8, respectively.

### Organic Substances

Some of the soil samples were enriched with the following organic substances: cellulose, sucrose, and compost, in the amounts of 0 and 9 g kg^−1^ dm of soil. The choice of these two doses was dictated by our previous experiments. Another consideration was to supply microorganisms with an optimal amount of carbon. Sucrose used for the experiment was provided by Chempur and cellulose was made by Acros Organics. The compost from Kommunalservice Vornkahl Polska was predominantly composed of waste from agriculture, horticulture, fisheries, forestry, and hunting as well as food and timber processing. The compost satisfies the European Union norms and is attested for use in agriculture, soil remediation, and industries. The compost had the following properties: pH_KCl_, 7.6; hydrolytic acidity, 18.0 mmol(+) kg^−1^; total exchangeable bases, 464 mmol(+) kg^−1^; organic carbon content, 188 g kg^−1^; and total nitrogen content, 4.4 g kg^−1^.

### Experimental Design

The experiment was performed with three replications. Batches of the soil material were placed in 150-cm^3^ glass beakers and then polluted with the set doses of polycyclic aromatic hydrocarbons. In order to enrich soil with nitrogen, each soil sample was added ammonium nitrate (NH_4_NO_3_) in the amount of 100 mg kg^−1^ dm of soil. In addition, organic substances were introduced to soil samples, after which all the components were carefully mixed and filled up with distilled water to 50 % of capillary water capacity. The soil material, prepared as explained above, was stored in an incubator at constant temperature of 25 °C, monitored nonstop, with no access to light, for 4, 8, and 16 weeks. During the whole experiment, the soil moisture content was monitored and maintained at a constant level of 50 % of capillary water capacity, replenishing any loss with demineralized water.

### Determination of Arylsulphatase Activity

After a set of time period (4, 8, or 16 weeks), soil samples were submitted to the determination of arylsulphatase activity according to the method by Alef and Nannipieri (1998). The determinations were performed with three replications. Fresh soil (2 g) together with 4 cm^3^ of 0.5 M acetate buffer and 1 cm^3^ of 0.02 M solution of potassium 4-nitrophenyl sulfate was incubated at 37 °C for 1 h. Next, 25 cm^3^ of distilled water was added to each sample and the whole contents of the beaker were filtered. Subsequently, 6 cm^3^ of the filtrate was sampled and mixed with 4 cm^3^ of 0.5 M sodium hydroxide. The absorbance of 4-nitrophenol thus produced was measured on an Aquarius CE7500 spectrophotometer (manufactured by CECIL Instruments) at a wavelength of 420 nm. The results were presented in millimoles PNP per kilogram decimeter per hour.

### Determination of Soil Resistance and Resilience Indices

Values of soil resistance (RS) and resilience (RL) indices were derived from the measured activity of arylsulphatase: the RS was calculated after 4 weeks of incubation, and the ability of soil to recover from the pollution (RL) was computed after 16 weeks of exposure to PAHs. The indices were derived from the formulas suggested by Orwin and Wardle (2004):

$$ \mathrm{RS}\left({t}_0\right)=1-\frac{2\left|{D}_0\right|}{\left({C}_0+\left|{D}_0\right|\right)} $$

where *D*_0_ is the difference between the control sample, assigned the symbol *C*_0_, and polluted soil samples after 4-week incubation (*t*_0_). Values of the RL index were calculated from the following formula:

$$ \mathrm{RL}\;\mathrm{at}\;{t}_x=\frac{2\left|{D}_0\right|}{\left(\left|{D}_0\right|+\left|{D}_x\right|\right)}-1 $$

where *D*_0_ was the same as above, while *D*_*x*_ means the difference between the control and polluted soil after 16-week incubation.

### Statistical Analysis

The research results were processed statistically, using determinations of homogenous groups according to Tukey’s test at significance level *p* = 0.01. The proportions of explained variance were calculated by multi-factorial variance analysis, employing the eta-square method. The principal component analysis (PCA) was applied to evaluate the effect of PAHs on the activity of arylsulphatase. All statistical computations were executed with a Statsitica 9.1 (StatSoft 2010) package. Correlation coefficients between the degree of soil pollution by PAHs and the activity of arylsulphatase were calculated in MS Excel.

## Results

### Activity of Arylsulphatase in Soil Contaminated with PAHs

The experimental results showed that soil contamination with PAHs in soil amended with organic substances such as cellulose, sucrose, and compost differentiated the activity of arylsulphatase in loamy sand (Tables 1, 2, 3, and 4). In the control series, all tested polycyclic aromatic hydrocarbons stimulated the activity of the enzyme, and their effect grew stronger as the soil contamination degree with naphthalene (Table 1), phenanthrene (Table 2), anthracene (Table 3), and pyrene (Table 4) rose higher.

**Table 1:** Activity of arylsulphatase in soil contaminated with naphthalene (mmol PNP kg^−1^ dm soil h^−1^)

Dose (mg kg^−1^ dm soil)	Type of organic substances			
	Control	Cellulose	Sucrose	Compost
4 weeks				
0	0.397 a^a^	0.802 cd	0.743 c	1.147 g
1,000	0.486 ab	0.858 cde	0.808 cd	1.034 fg
2,000	0.515 ab	0.860 cde	0.881 de	0.978 ef
4,000	0.554 b	0.888 de	0.921 def	0.904 def
Average	0.488 ab	0.852 cde	0.838 cd	1.016 fg
*r*	0.923^*^	0.988^*^	0.927^*^	−0.986^*^
8 weeks				
0	0.324 a	0.757 h	0.572 de	0.351 ab
1,000	0.579 de	0.880 i	0.637 efg	0.404 abc
2,000	0.588 def	0.914 jk	0.674 efgh	0.450 bc
4,000	0.701 fgh	1.019 k	0.723 gh	0.479 cd
Average	0.548 de	0.893 i	0.652 efg	0.421 bc
*r*	0.886^*^	0.972^*^	0.993^**^	0.992^**^
16 weeks				
0	0.313 a	0.743 e	0.570 c	0.303 a
1,000	0.559 c	0.892 f	0.722 de	0.306 a
2,000	0.654 d	1.078 g	0.737 e	0.369 ab
4,000	0.753 e	1.285 h	0.791 e	0.397 b
Average	0.570 c	1.000 g	0.705 d	0.344 ab
*r*	0.921^*^	0.948^*^	0.898^*^	0.771^*^

^a^In columns and rows, in individual terms, homogeneous groups are followed by the same letter

* *r* at level *p* = 0.05

** *r* at level *p* = 0.01

**Table 2:** Activity of arylsulphatase in soil contaminated with phenanthrene (mmol PNP kg^−1^ dm soil h^−1^)

Dose (mg kg^−1^ dm soil)	Type of organic substances			
	Control	Cellulose	Sucrose	Compost
4 weeks				
0	0.397 a^a^	0.802 bc	0.743 b	1.147 e
1,000	0.399 a	0.906 cd	0.887 cd	0.975 d
2,000	0.403 a	0.968 d	0.900 cd	0.974 d
4,000	0.409 a	0.989 d	0.938 cd	0.908 cd
Average	0.402 a	0.916 cd	0.867 cd	1.001 de
*r*	0.993^**^	0.854^*^	0.968^*^	−0.996^**^
8 weeks				
0	0.324 a	0.757 h	0.572 de	0.351 ab
1,000	0.612 ef	0.918 i	0.630 ef	0.389 abc
2,000	0.668 efg	0.913 i	0.656 efg	0.441 bc
4,000	0.706 gh	0.989 i	0.758 h	0.468 cd
Average	0.577 de	0.894 hi	0.654 efg	0.412 bc
*r*	0.822^*^	0.973^*^	0.902^*^	0.935^*^
16 weeks				
0	0.313 a	0.743 fg	0.570 cd	0.303 a
1,000	0.553 c	0.867 i	0.682 ef	0.372 ab
2,000	0.642 de	0.980 j	0.778 gh	0.396 b
4,000	0.746 fg	1.103 k	0.833 hi	0.443 b
Average	0.563 c	0.923 ij	0.716 fg	0.378 ab
*r*	0.925^*^	0.972^*^	0.988^*^	0.983^*^

^a^Explanation as in Table 1

**Table 3:** Activity of arylsulphatase in soil contaminated with anthracene (mmol PNP kg^−1^ dm soil h^−1^)

Dose (mg kg^−1^ dm soil)	Type of organic substances			
	Control	Cellulose	Sucrose	Compost
4 weeks				
0	0.417 a^a^	0.806 cd	0.743 bc	1.140 e
1,000	0.832 cd	0.845 cd	0.816 cd	0.880 d
2,000	0.840 cd	0.863 d	0.903 d	0.879 d
4,000	0.910 d	0.891 d	0.908 d	0.671 b
Average	0.750 bc	0.851 cd	0.843 cd	0.892 d
*r*	0.785^*^	0.914^*^	0.953^*^	−0.895^*^
8 weeks				
0	0.323 a	0.759 g	0.581 de	0.348 a
1,000	0.492 bc	0.775 g	0.587 de	0.450 b
2,000	0.532 cd	0.784 g	0.624 ef	0.454 b
4,000	0.574 de	0.813 g	0.674 f	0.457 bc
Average	0.480 bc	0.783 g	0.616 e	0.427 b
*r*	0.870^*^	0.869^*^	0.970^*^	0.598^*^
16 weeks				
0	0.324 a	0.746 de	0.582 c	0.313 a
1,000	0.545 c	0.766 ef	0.688 d	0.312 a
2,000	0.582 c	0.877 gh	0.733 de	0.370 a
4,000	0.715 de	0.914 h	0.825 fg	0.449 b
Average	0.542 c	0.826 fg	0.707 d	0.361 a
*r*	0.936^*^	0.862^*^	0.915^*^	0.896^*^

^a^Explanation as in Table 1

**Table 4:** Activity of arylsulphatase in soil contaminated with pyrene (mmol PNP kg^−1^ dm soil h^−1^)

Dose (mg kg^−1^ dm soil)	Type of organic substances			
	Control	Cellulose	Sucrose	Compost
4 weeks				
0	0.417 a^a^	0.806 bc	0.743 b	1.140 j
1,000	0.818 bcd	0.974 fgh	0.860 cde	1.011 ghi
2,000	0.841 bcde	1.034 hi	0.910 def	0.973 fgh
4,000	0.885 cdef	1.099 ij	0.934 efg	0.845 cde
Average	0.740 b	0.978 fgh	0.862 cde	0.992 fgh
*r*	0.771^*^	0.957^*^	0.995^**^	−0.941^*^
8 weeks				
0	0.323 a	0.759 efg	0.581 d	0.348 a
1,000	0.456 bc	0.763 efg	0.699 e	0.429 b
2,000	0.477 bc	0.776 fg	0.742 ef	0.481 bc
4,000	0.481 bc	0.819 g	0.758 efg	0.501 c
Average	0.434 bc	0.779 fg	0.695 e	0.440 bc
*r*	0.762^*^	0.578^*^	0.694^*^	0.989^**^
16 weeks				
0	0.324 a	0.746 fg	0.582 d	0.313 a
1,000	0.488 c	0.818 gh	0.637 de	0.349 ab
2,000	0.576 d	0.856 hi	0.703 ef	0.415 bc
4,000	0.759 fg	0.937 i	0.747 fg	0.429 bc
Average	0.537 cd	0.839 h	0.667 e	0.377 b
*r*	0.989^**^	0.999^**^	0.979^*^	0.989^**^

^a^Explanation as in Table 1

Irrespectively of the incubation time, the strongest stimulating effect was produced by anthracene, and the weakest one was exerted by phenanthrene. When these two PAHs had been applied to soil in the amount of 4,000 mg kg^−1^ dm of soil, the activity of arylsulphatase increased by an average of 49.6 and 38.3 %, respectively. In the series with cellulose and sucrose, the activity of arylsulphatase increased with a rising degree of pollution with any of the tested PAHs. In the samples with compost, the activity of arylsulphatase was stimulated by doses of naphthalene, phenanthrene, anthracene, and pyrene after 8 and 16 weeks of incubation. After 4-week incubation, the activity of arylsulphatase decreased parallel to the growing degree of soil contamination with the PAHs.

The factor that significantly affected the arylsulphatase activity was the duration of soil incubation, especially in samples with anthracene and pyrene, where the incubation period as a variable explained 32.05 and 39.12 % of variance, respectively (Table 5). The activity of arylsulphatase was decreasing with time until the 8 weeks of incubation. After 16 weeks, a slight increase in the activity of arylsulphatase versus the 8-week incubation period was noticed. Regardless of the degree of soil pollution with PAHs and type of organic substance added to soil, the soil samples with naphthalene were observed to demonstrate a 27 % lower activity of arylsulphatase after 8 weeks of incubation compared to the 4-week incubation period. An analogous situation developed in soil samples with phenanthrene, anthracene, and pyrene, where the activity of arylsulphatase declined by 25.5, 45, and 52 %, respectively.

**Table 5:** Percentage of variable factors on the basis of the activity of arylsulphatase

Variables	PAHs			
	Naphthalene	Phenanthrene	Anthracene	Pyrene
Dose	9.14	8.54	7.02	8.66
Term	9.69	9.64	32.05	39.12
OS	36.91	40.27	26.60	27.47
Dose · term	3.17	2.88	1.75	0.82
Dose · OS	4.03	2.91	9.11	4.47
Term · OS	34.10	32.68	16.01	14.06
Dose · term	1.82	1.91	6.49	4.67
Error	1.14	1.18	0.96	0.73

*OS* organic substance

Determination of the proportion of explained variance, with the *η* ^2^ factor and the ANOVA method, revealed that the activity of arylsuphatase in the PAH-contaminated soil was mostly determined by soil amendment with organic substances. In samples exposed to naphthalene pressure, the contribution of this variable to explained variance reached about 36.91 %; in the case of phenanthrene, it went up to 40.27 %; in anthracene pollute soil, it equalled 26.60 %; and in soil samples with pyrene, it was determined at 27.47 % (Table 5). The application of cellulose, sucrose, and compost to soil had a positive effect on the activity of arylsulphatase. Irrespectively of the degree of soil contamination with PAHs and incubation time, it has been shown that the activity of arylsulphatase in samples polluted with naphthalene, phenanthrene, anthracene, and pyrene when enriched with cellulose was higher by 1.7-, 1.7-, 1.4-, and 1.5-fold, respectively; when sucrose was added to soil, the arylsuphatase activity was 1.4-, 1.4-, 1.2-, and 1.3-fold higher. Addition of compost to soil raised the activity of arylsulphatase in samples polluted with naphthalene by 1.1-fold, with phenanthrene by 1.2-fold and with pyrene by 1.05-fold; reversely, in samples polluted with anthracene and treated with sucrose, a slight decrease in arylsulphatase activity versus the control appeared. Addition of organic substances lessened long-term effects of PAHs. The soil application of cellulose, sucrose, and compost resulted in the stabilization of arylsulphatase activity in the analyzed time period. Regardless the applied dose or date of determinations, the stimulating effect of organic substances can be put in the following order: cellulose > sucrose > compost, with cellulose raising the activity of the enzyme by an average of 58 %, sucrose by 33 %, and compost by 6 % against the control.

### Resistance of Soil (RS) to Contamination with PAHs

**Fig. 1:**
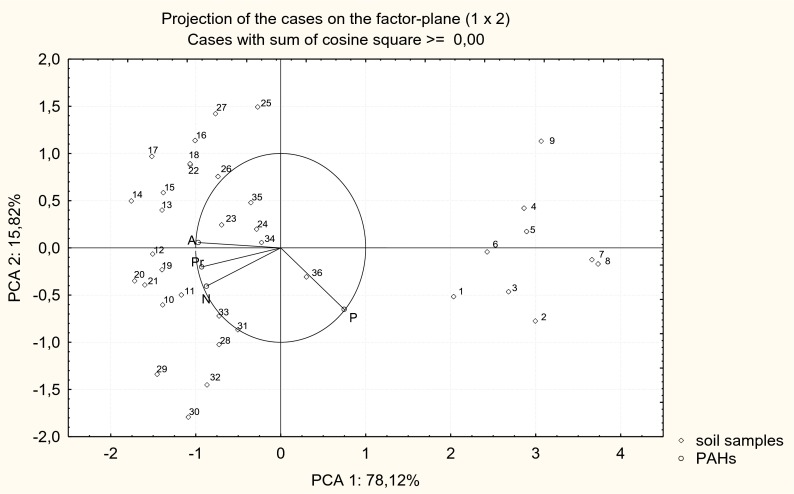
Resistance of soil contaminated with PAHs presented by PCA method. Vectors defined analyzed variables (*N* naphthalene, *P* phenanthrene, *A* anthracene, *Pr* pyrene), and points—soil samples with organic substances. Explanations: *1–9* soil samples with no organic substances, contaminated with PAHs respectively in the amounts of 1,000 (*1–3*), 2,000 (*4–6*), and 4,000 mg kg^−1^ dm soil (*7–9*); *10–18* soil samples with cellulose, contaminated with PAHs, respectively, in the amounts of 1,000 (*10–12*), 2,000 (*13–15*), and 4,000 mg kg^−1^ dm soil (*16–18*); *19–27* soil samples with sucrose, contaminated with PAHs, respectively, in the amounts of 1,000 (*19–21*), 2,000 (*22–24*), and 4,000 mg kg^−1^ dm soil (*25–27*); *28–36* soil samples with compost, contaminated with PAHs, respectively, in the amounts of 1,000 (*28–30*), 2,000 (*31–33*), and 4,000 mg kg^−1^ dm soil (*34–36*)

The resistance of loamy sand to contamination of naphthalene, phenanthrene, anthracene, and pyrene was varied, as shown by data in Table 6 and Fig. 1, which depicts the position of dependent variables (PAHs) and points representing the resistance of loamy sand according to the activity of arylsulphatase. The vectors of naphthalene, anthracene, and pyrene are more strongly inclined towards the main horizontal axis, which explains over 78 % of the total correlation of the set of data. The figure shows strong correlations between naphthalene, anthracene, and pyrene. The points illustrating the resistance in soil without organic substances are located further away from the vectors of variables. This proves that the soil not amended with cellulose, sucrose, or compost was characterized by a weaker resistance to PAHs than the one fertilized with the said substances.

**Table 6:** Resistance in soil (RS) contaminated with PAHs

Dose (mg kg^−1^ dm soil)	Type of organic substances			
	Control	Cellulose	Sucrose	Compost
Naphthalene				
1,000	0.611 cd	0.804 ab	0.837 ab	0.878 a
2,000	0.521 de	0.799 ab	0.685 bcd	0.791 abc
4,000	0.413 f	0.744 abc	0.613 cd	0.690 bcd
Average	0.515 de	0.782 abc	0.711 bc	0.786 abc
Phenanthrene				
1,000	0.884 a	0.709 bcde	0.672 cde	0.786 abcd
2,000	0.826abc	0.599 e	0.649 de	0.785 abcd
4,000	0.872 ab	0.566 e	0.582 e	0.695 cde
Average	0.861 ab	0.624 e	0.635 de	0.756 bcd
Anthracene				
1,000	−0.021 d	0.904 a	0.808 a	0.614 b
2,000	−0.029 d	0.861 a	0.636 b	0.612 b
4,000	−0.105 d	0.805 a	0.625 b	0.408 c
Average	−0.051 d	0.857 a	0.687 b	0.545 b
Pyrene				
1,000	−0.004 e	0.652 bc	0.717 ab	0.775 a
2,000	−0.031 e	0.554 cd	0.621 bc	0.726 ab
4,000	−0.080 e	0.464 d	0.581 cd	0.575 cd
Average	−0.038 e	0.556 cd	0.639 bc	0.692 bc

In columns and rows, for individual PAHs, homogeneous groups are followed by the same letter

Samples with added cellulose, sucrose, and compost are closer to vectors of variables, which means that each of the applied organic substances reduced the negative effect of naphthalene, phenanthrene, anthracene, and pyrene. The positive influence of organic substances can be put in the following order: cellulose > compost > sucrose. Regardless of the type of a polycyclic aromatic hydrocarbon, introduction of cellulose to soil raised its average resistance by 2.18-fold compared to the control. Compost improved soil resistance by 2.16-fold and sucrose by 2.07-fold.

### The Resilience of Loamy Sand

Values of the resilience (RL) index for loamy sand were determined after 16 weeks of incubation of soil samples. Polycyclic aromatic hydrocarbons in the control series and in the series with added organic substances produced lasting changes in loamy sand. This was confirmed by low average values of the RL index (Table 7), especially for the soil samples from the control series subjected to contamination with phenanthrene, where the mean RL was −0.83. The highest average value of the RL index (0.24) was calculated for soil with pyrene.

When cellulose was added to loamy sand, the average values of RL were higher versus the control only in samples polluted with phenanthrene and pyrene. The samples with naphthalene and anthracene were observed to yield lower mean RL values than in the control. In soil samples with sucrose polluted with naphthalene and phenanthrene, the average values of the RL index were higher, unlike in soil with anthracene or pyrene, where they were lower than in the control samples. In the series with compost, the average RL values higher than the control were recorded in soil submitted to pressure of all of the tested PAHs (Table 7).

**Table 7:** Resilience of soil contaminated with PAHs

Dose (mg kg^−1^ dm soil)	Type of organic substances			
	Control	Celullose	Sucrose	Compost
Naphthalene				
1,000	−0.426 cd^a^	−0.288 cd	−0.394 cd	0.690a
2,000	−0.454 cd	−0.594 cd	−0.089 bc	0.302 ab
4,000	−0.448 cd	−0.655 d	−0.110 bc	0.364 ab
Average	−0.443 cd	−0.512 cd	−0.198 c	0.452 b
Phenanthrene				
1,000	−0.811 d	0.019 abc	0.141 ab	0.304 a
2,000	−0.789 d	−0.106 bc	−0.142 bc	0.164 ab
4,000	−0.881 d	−0.256 c	−0.146 bc	0.167 ab
Average	−0.827 d	−0.114 bc	−0.049 bc	0.212 ab
Anthracene				
1,000	0.305 abc	0.255 abcd	−0.160 cd	0.870 a
2,000	0.242 abcd	−0.371 d	0.040 bcd	0.608 ab
4,000	0.117 bcd	−0.323 cd	−0.177 cd	0.532 ab
Average	0.221 bc	−0.146 cd	−0.099 bcd	0.670 ab
Pyrene				
1,000	0.420 ab	0.405 ab	0.367 ab	0.530 a
2,000	0.252 ab	0.357 ab	0.100 ab	0.238 ab
4,000	0.042 b	0.217 ab	0.079 ab	0.418 ab
Average	0.238 ab	0.327 ab	0.205 ab	0.395 ab

^a^Explanation as in Table 6

## Discussion

### The Effect of PAHs on Arylsulphatase Activity

The activity of soil enzymes is one of the approved parameters used for evaluation of the quality of soil polluted with organic compounds, including polycyclic aromatic hydrocarbons. Arylsulphatase is one of the enzymes responsible for functions of the mineralization factor and sulfur cycling in soil. Its activity depends on a number of factors, including amounts of introduced pollutants (Kang and Freeman 1999). This assertion is supported, for instance, by the results presented herein, which point to a gradual increase in the activity of arylsulphatase parallel to an increased degree of soil contamination with naphthalene, phenanthrene, anthracene, and pyrene. Contrary results were obtained by Gianfreda et al. (2005) who noticed a decreasing activity of this enzyme as the quantities of PAHs in soil rose. Stimulation of the activity of soil enzymes, including arylsulphatase, may be attributed to the properties of polycyclic aromatic hydrocarbons and especially the ones connected with specific structures of molecules of particular PAHs. In a study by Smreczak and Maliszewska-Kordybach (2003) on the bioavailability of the PAH fraction in soils, the researchers reported a share of hydrocarbons composed of four benzene rings being from 1.5- to 11-fold higher than hydrocarbons containing five or six benzene rings in a molecule. It is assumed that two- to four-ringed aromatic hydrocarbons (naphthalene, phenanthrene, anthracene, and pyrene) are more readily absorbed by microorganisms, which in turn translates into a higher enzymatic activity (Baran et al. 2004; Boopathy 2000).

The experiment presented herein has proven the significance of the incubation time of soil samples polluted by particular PAHs in terms of the activity of arylsulphatase. Both in the authors’ own research and in a study performed by Serrano et al. (2009), which focused on the biological activity of soil in response to its pollution with crude oil, the activity of arylsulphatase was observed to have diminished relative to its activity in unpolluted soil during the first 50 days of the experiment. On a later date, the cited authors found a 42.3 % higher activity of this enzyme compared to the control soil, with the result persisting until the experiment was terminated. Our investigations have shown a slight increase in the activity of arylsulphatase after 16 weeks of soil incubation as compared to the results achieved after 8 weeks of the experiment. In all probability, the reason was the decomposition of organic substances by active microorganisms, which gradually released carbon from naphthalene, phenanthrene, anthracene, and pyrene. While adjusting to new environmental conditions and expressing a higher demand for metabolic processes, microorganisms may turn to xenobiotic substances as an additional source of carbon and energy (Tejada et al. 2008; Wang et al. 2007; Zafra et al. 2014).

The content of polycyclic aromatic hydrocarbons in soils may fluctuate in response to abiotic events (sorption on mineral and organic colloids, evaporation with water evaporating from soil) and biological changes (microbial breakdown by bacteria, actinomycetes, and fungi). The ability of some microorganisms to decompose PAHs is often inhibited by some unfavorable environmental conditions, which is why introduction of additional organic substances to soil is extremely important, the fact confirmed in the authors’ own studies and by other researchers (Wyszkowska and Wyszkowski 2010; Tejada et al. 2008; Cai et al. 2007; Sayara et al. 2010). Stimulation of the biochemical activity by different organic substances correlates closely to providing microorganisms with an additional source of carbon and energy. One of the most frequently tested organic substances is cellulose, whose role in the sorption of PAHs is small, as proven by studies carried out by Wang et al. (2007) and Jonker (2008). Nevertheless, the application of cellulose in the amount of 9 g kg^−1^ dm of soil had a positive effect on the activity of arylsulphatase. Wyszkowska et al. (2006) also noticed elevated activities of soil enzymes in petrol-polluted soil enriched with oat straw. Another substance tested herein was sucrose, an easily decomposable and absorbable disaccharide, which aids the development of microorganisms, providing them with a good source of energy.

The increased activity of microorganisms in soil exposed to pressure by PAHs and supplemented with glucose has been reported by Mühlbachovă (2008). Compost used in the present experiment had a positive influence on the activity of arylsulphatase, and its stimulating effect has been implied by other authors as well (Cai et al. 2007; Sayara et al. 2010, 2011). From an increased activity of arylsulphatase, Scelza et al. (2007) concluded that compost had a positive effect on soils polluted with phenanthrene, which may have been a reflection of an increased number of autochthonous microorganisms.

### Soil Resistance and Resilience in Soil Contaminated with PAHs

The resistance of soil and its ability to recover the equilibrium state, which is soil resilience, are essential quality components and key indicators of the homeostasis between pressure caused by pollutants and the capacity of soil for regeneration. The RS index is determined by the magnitude of changes caused by pollutants introduced to the soil environment, while the soil recovery to the equilibrium state (RL) is the response to the previously occurring stresses (Orwin and Wardle 2004). Among the most substantial factors affecting soil resistance and resilience, researchers mention the type of soil and plants, climate, land use, and the extent of external interference. Interactions between the RS, RL, and duration of the contamination are complicated and case specific. The concept of studying soil resistance and resilience in relation to the activity of soil enzymes has been presented in papers dedicated to soil contamination with heavy metals (Wyszkowska et al. 2010, 2013). The issue of the resistance of soil polluted with PAHs is less often raised in literature (Lipińska et al. 2013, 2014). Most of the RS index values are within the range of −1 to 1, with −1 meaning that the effect of analyzed contamination on soil appeared in 200 %, 0 in 100 %. When the RS equals 1, soil is fully tolerant to a given contaminant, which is zero effect of contamination appeared. The RS index values obtained in our experiment implied that the soil material with pyrene was distinguished by the highest resilience, with the mean value of the RL index 0.23. The closer the RL value to 1, the higher the soil’s capacity to counteract external factors. Low values of the RL index imply the long-term effect of polycyclic aromatic hydrocarbons in loamy sand.

The above results and assumptions constitute one type of research pursued in order to expand the concept of soil resistance (RS) and resilience (RL) based on the activity of enzymes determined in soil contaminated with PAHs.

## Conclusions

Soil contamination with naphthalene, phenanthrene, anthracene, and pyrene in the amounts of 0–4,000 mg kg^−1^ dm of soil stimulated the activity of arylsulphatase, which significantly depended on the dose of PAHs, incubation time, and type of organic substances introduced to soil. The highest stimulating effect in the control series was determined for anthracene, and the weakest one for phenanthrene. The activity of arylsulphatase continued to decrease until the 8th week of the experiment, after which it slightly increased. All of the applied organic substances (cellulose, sucrose, compost) had a positive effect on the activity of arylsulphatase, with the highest stimulating influence attributed to cellulose, and the weakest one to compost. The lowest resistance (RS) was found in soil contaminated with PAHs and not amended with any organic substance; the highest RS was determined in soil samples with applied sucrose and compost. Based on the RL values in the control series, it was concluded that soil contaminated with pyrene most rapidly recovered the equilibrium state, while soil subjected to the pressure of phenanthrene took the longest to recover.
